# Cardiovascular Changes in Atherosclerotic ApoE-Deficient Mice Exposed to Co60 (γ) Radiation

**DOI:** 10.1371/journal.pone.0065486

**Published:** 2013-06-20

**Authors:** Prem Kumarathasan, Renaud Vincent, Erica Blais, Anu Saravanamuthu, Pallavi Gupta, Heather Wyatt, Ronald Mitchel, Mohammed Hannan, Akilesh Trivedi, Stewart Whitman

**Affiliations:** 1 Analytical Biochemistry and Proteomics Laboratory, Environmental Health Science and Research Bureau, Healthy Environments and Consumer Safety Branch, Health Canada, Ottawa, Ontario, Canada; 2 Inhalation Toxicology Laboratory, Environmental Health Science and Research Bureau, Healthy Environments and Consumer Safety Branch, Health Canada, Ottawa, Ontario, Canada; 3 Radiological Protection Research and Instrumentation Branch, Atomic Energy Canada Limited, Chalk River Laboratories, Chalk River, Ontario, Canada; 4 Radiation Protection Bureau, Healthy Environments and Consumer Safety Branch, Health Canada, Ottawa, Ontario, Canada; 5 Department of Pathology and Laboratory Medicine, University of Ottawa, Ottawa, Ontario, Canada; Kagoshima University Graduate School of Medical and Dental Sciences, Japan

## Abstract

**Background:**

There is evidence for a role of ionizing radiation in cardiovascular diseases. The goal of this work was to identify changes in oxidative and nitrative stress pathways and the status of the endothelinergic system during progression of atherosclerosis in ApoE-deficient mice after single and repeated exposure to ionizing radiation.

**Methods and Results:**

B6.129P2-ApoE tmlUnc mice on a low-fat diet were acutely exposed (whole body) to Co60 (γ) (single dose 0, 0.5, and 2 Gy) at a dose rate of 36.32 cGy/min, or repeatedly (cumulative dose 0 and 2 Gy) at a dose-rate of 0.1 cGy/min for 5 d/wk, over a period of 4 weeks. Biological endpoints were investigated after 3–6 months of recovery post-radiation. The nitrative stress marker 3-nitrotyrosine and the vasoregulator peptides endothelin-1 and endothelin-3 in plasma were increased (p<0.05) in a dose-dependent manner 3–6 months after acute or chronic exposure to radiation. The oxidative stress marker 8-isoprostane was not affected by radiation, while plasma 8-hydroxydeoxyguanosine and L-3,4-dihydroxyphenylalanine decreased (p<0.05) after treatment. At 2Gy radiation dose, serum cholesterol was increased (p = 0.008) relative to controls. Percent lesion area increased (p = 0.005) with age of animal, but not with radiation treatment.

**Conclusions:**

Our observations are consistent with persistent nitrative stress and activation of the endothelinergic system in ApoE−/− mice after low-level ionizing radiation exposures. These mechanisms are known factors in the progression of atherosclerosis and other cardiovascular diseases.

## Introduction

Exposure of humans to ionizing radiation can either be of environmental or occupational nature, or through diagnosis and treatment of diseases. Although ionizing radiation is investigated mainly in terms of induction of cancer, somatic mutations in exposed individuals can also lead to other maladies, and heritable mutations are implicated in disease risk in future generations [1]. While radiotherapy has been successful in increasing the survival outcome of cancer patients, it is also associated unfortunately with serious health outcomes [2]. For example, radiation-induced secondary carcinoma has been shown to occur many years following completion of radiation therapy [2]–[4]. Radiation can induce DNA damage, changes in gene expression, cell cycle arrest, and apoptotic cell death in the directly exposed cells [1], [4]. It is known that radiation can also mediate effects in neighbouring un-irradiated cells or in naïve cells that received distress signals from the directly exposed cells, a phenomenon known as bystander effect [5]–[8].

Ionizing radiation is also implicated in precipitating other non-cancer diseases, such as cardiovascular diseases, birth defects, and ocular diseases [1], [9]–[12]. Radiation-induced cardiovascular diseases are known causes of mortality and morbidity in patients undergoing radiotherapy, in radiologists, and in nuclear industry workers [13]–[17]. For example, survivors of Hodgkin’s disease and breast cancer who received radiotherapy have a higher mortality from cardiovascular diseases than the general population [9], [18]. Other conditions associated with direct effects of radiation on the heart include pericarditis, myocardial fibrosis, coronary artery disease, valvular disease, conduction system disease/arrhythmias, autonomic dysfunction, dilated or restricted cardiomyopathy, and vascular changes [9]. Radiation can have additional indirect effects on heart function by affecting structures in the neck and the chest, which the heart depends upon [9]. Epidemiological studies on A-bomb survivors have shown significant increase in hypertension and myocardial infarction [10]–[12]. Furthermore, there are reports relating radiation-induced psycho-social factors to subsequent effects on the cardiovascular system [19].

Several pathologies, including cardiovascular diseases such as hypertension and atherosclerosis, are associated with oxidative stress [20]. Oxidative stress *in vivo* is known to be associated with inflammatory pathways and endothelial injury especially in cardiovascular disorders [21]–[23]. Meanwhile, exposure to ionizing radiation generates free radicals *in vivo,* and this can impact on the etiology of cardiovascular disease. In addition, previous reports from *in vitro* and *in vivo* radiation exposure studies suggest oxidative stress as well as epigenetic changes as potential mechanisms underlying bystander effects [24]–[27]. Radiation-induced cardiovascular diseases exhibit characteristics of such bystander effects.

While exposure to acute low dose radiation can reduce atherosclerosis lesions in ApoE-deficient mice, an effect that can be interpreted as protective [28], it also can increase delayed cardiac fibrosis due to a pro-inflammatory state [29]. We hypothesized that the investigation of upstream events in the biological response cascade may shed some light into the dose-response relationship and the dynamics of effects. We exposed ApoE-deficient mice to radiation acutely (36.32 cGy/min dose-rate) and chronically (0.1 cGy/min dose-rate) and investigated changes in biomarkers of oxidative stress, nitrative stress, and endothelial dysfunction following several months of recovery post-radiation. Our results reveal that persisting biochemical changes can be measured in plasma after 3–6 months, revealing a marked increase in the rate of reactive nitrogen species formation accompanied by a doubling of the steady-state concentration of the potent vasoactive and mitogenic peptide endothelin-1.

## Methods

### Animals

Female mice homozygous for the Apoe tmlUnc mutation (B6.129P2-Apoe tmlUnc) were obtained from Jackson Labs (Maine, U.S.A). These mice display marked atherosclerosis when fed a high fat diet and moderate atherosclerosis when fed a normal/low fat diet. The mice in this study were fed a low fat diet to observe the effects of radiation on the progression of atherosclerosis. Health monitoring during and at the end of the study indicated no change in specific pathogen-free status.

### Ethics

Protocols were reviewed and approved by the AECL and Health Canada Animal Care Committees. All housing, handling and experimental procedures were conducted in strict accordance to the principles and procedures set forth in the guidelines of the Canadian Council on Animal Care (http://www.ccac.ca/en_/standards/guidelines).

### Material

Dulbecco’s phosphate-buffered saline (PBS, calcium and magnesium free), ethylenediaminetetra acetic acid (EDTA), diethylenetriaminepentaacetic acid (DETPA), phenylmethylsulfonyl fluoride (PMSF), trifluoroacetic acid (TFA), 3,4-dichloroisocoumarin sodium acetate, trisodium salt of citric acid, octanesulfonic acid sodium salt (OSA), trizma hydrochloride, trizma base, NADPH, sodium hydroxide, nitrate reductase, sodium nitrite, potassium nitrate, molecular weight cut-off filters (10 and 30 kDa) and standards of p-tyrosine, 3-nitrotyrosine, L-3,4-dihydroxyphenylalanine (L-DOPA), 8-hydroxydeoxyguanosine and endothelins (BET-1, ET-1, ET-2, ET-3) were purchased from Sigma (St. Louis, MO, USA). Analytical reagent-grade NaCl, and HCl were from BDH (Toronto, Canada). Reagent-grade acetone, acetonitrile, and methanol were from commercial suppliers. Butylated hydroxytoluene (BHT) was from United States Biochemical Corporation (Cleveland, OH, USA). Deionized water (DI water) was obtained from a super-Q plus high purity water system (Millipore, Bedford, MA, USA). UHP-grade compressed nitrogen was supplied by Matheson Gas products (Whitby, Canada). Amber glass vials and screw caps with septa were purchased from Chromatographic specialities, Inc. (Brockville, ON). 2, 3-diaminonaphthalene was obtained from Molecular Probes (Eugene, OR). C18 Sep Pak Cartridges were obtained from Waters (Milford, MA). EIA kit for 8-isoprostane analysis was purchased from Cayman chemicals (Ann Arbor, Michigan, USA).

### Exposure to Radiation

Mice weighing 18–22 g were divided into 5 groups and exposed (whole body) acutely (single exposure) or chronically (repeated exposures) to Co60 (γ) radiation as follows. The mice were 2 months old at start of radiation exposures and were at the early stage of atherosclerosis [28]. *Acute Exposure*: *Group 1,* 0 Gy (no radiation); *Group 2,* 0.5 Gy (36.32 cGy/min for 75.8 seconds); *Group 3,* 2 Gy (36.32 cGy/min for 323.6 seconds). *Chronic Exposure*: *Group 4,* 0 Gy (no radiation); *Group 5,* 2 Gy (0.1 cGy/min, 10cGy per day, 5 days a week for 4 weeks for a total dose of 2 Gy). The mice were sacrificed after three to six months recovery after the last radiation exposure [28], [29].

### Plasma and Serum Sample Preparation

Animals were terminated by sodium pentobarbital and blood samples were collected upon anaesthesia from the right ventricle by cardiac puncture using heparinised syringes. A portion of the blood sample was transferred into a vacutainer tube containing the sodium salt of EDTA (10 mg/mL) and PMSF (1.75 mg/mL), mixed gently and placed on ice. The remaining portion was transferred into a serum collection tube, left at room temperature for the collection of serum as described before [28]. Whole blood samples stabilized with preservatives (EDTA, PMSF) were centrifuged at 2000 rpm for 10 min to obtain plasma [30].

### Collection of Tissue

Mice were perfused with PBS via a cannula placed in the left ventricle with perfusate drained from severed right atrium and tissue collection was carried out as described previously [31], [32]. Briefly, hearts were separated from the aorta at the base, embedded in optimum cutting temperature (OCT) medium, and snap frozen on a metal plate that was cooled with liquid nitrogen. Aorta was fixed in 4% paraformaldehyde solution.

### Plasma 8-Isoprostane

Aliquots of plasma (250 µL) samples were stabilized with diethylenetriaminepentaacetic acid (DETPA) and butylated hydroxytoluene (BHT) to prevent any autoxidation for the 8-isoprostane analysis. These plasma samples were initially purified using the C18 Sep Pak cartridges (Waters) as described before [33]. Following this, the C-18 bound 8-isoprostane was eluted using methanol and was dried under N_2_ flow in amber glass vials. These purified plasma samples were then analyzed for 8-isoprostane using an enzyme immunoassay kit (EIA kit from Cayman chemicals).

### Markers of Protein Oxidation and Nitration

Markers of both reactive oxygen and nitrogen species generation were analyzed using the HPLC-EC array method [34]. In brief, 250 µL aliquots of plasma samples stabilized with DETPA and BHT were initially deproteinized by use of acidified acetone and were subsequently clarified using molecular weight cut-off filters (30 kDa). Nitrogen dried samples were then reconstituted with 100 µL of acidified water prior to analysis by HPLC-EC array technique. Initial separation of analytes were carried out on a LC-18 reversed phase column (25 cm length, 4.6 mm id, 5 µm particle size; Supelco, Oakville, ON) by isocratic elution using a citrate-acetate buffer (pH = 4.7) mobile phase containing OSA as the ion-pair reagent. Separated analytes were measured by coulometric array detection using a set of eight electrodes at different applied potentials.

### Circulating Endothelin Isoforms

This procedure was conducted as described before [30]. Briefly, aliquoted plasma samples (250 µL) were treated with 3,4-dichloroisocoumarin solution in isopropanol to prevent conversion of big ET-1 to ET-1 during sample processing. These samples were then deproteinized by acidified acetone, followed by clean up using molecular weight cut-off filters (30 kDa). Clarified samples were dried under a N_2_ flow and were reconstituted in phosphate buffered saline and were analyzed by a reversed phase HPLC-Fluorescence system. Initial separation of endothelin isoforms were carried out on a LC-318 column (25 cm length, 4.6 mm id, 5 µm particle size; Supelco, Oakville, ON) by gradient elution using water-acetonitrile mobile phase (A-30% acetonitrile (aq); B-90% acetonitrile (aq)) with 0.19% of TFA used as the ion-pair reagent. Analytes were measured by fluorescence detection at excitation and emission wavelengths of 240 nm and 380 nm, respectively.

### Plasma Nitrite

This method is based on thermolysis of all NO-related compounds *in vivo* such as nitrosyl metal complexes, nitrite/nitrate, S-nitroso compounds in plasma to nitrate, followed by enzymatic reduction to nitrite that was derivatized using a fluorescent probe for detection by fluorescence. Nitrite analysis was conducted following a previously reported procedure [30]. In essence, plasma samples (50 µL) treated with DETPA and BHT were thermolyzed at 86°C, cooled and filtered via 10 kDa molecular weight cut-off filters. These samples were treated with nitrate reductase from Sigma Chemical (St. Louis, MO) at room temperature for an hour, and were derivatized with 2,3 diaminonaphthalene from Molecular Probes (Eugene, OR). Fluorescence measurements were made at Ex λ = 360 nm; Em λ = 460 nm on a Cytofluor 2350 multiplate fluorescence detector (Millipore, Bedford, MA).

### Serum Cholesterol

Total cholesterol levels in serum samples were determined using a colorimetric assay (Wako Bioproducts, Richmond, VA) as described before [28]. Note that the mice were not starved prior to blood withdrawal for cholesterol to refrain from introducing additional stress on these ApoE-deficient mice with a progressing pathology.

### Quantification of Lesions

Quantification of the percent atherosclerotic lesion area covering the aortic arch in an en face preparation of the vessel was performed as described previously [31], [32]. Animals examined here were 19 weeks of age at termination for the acute exposure and 22 weeks of age for the chronic exposure.

### Statistics

Differences between treatment groups were determined by ANOVA (SigmaStat v3.5, SPSS Inc., Chicago, IL). For histopathology and cholesterol measurements, all animals (n = 15 per group) were studied after 3-month recovery and were all 5 months of age for the acute exposure, and 5.5 months of age for the chronic exposure. For all other biochemical endpoints, low plasma recovery required the use of animals after 3-month and 6-month recovery. Animals were age matched and the treatment groups within the acute exposure (0. 0.5, 2 Gy) or the chronic exposure (0, 2 Gy) remained balanced, although animals of the chronic study are on average 2–3 weeks older than the animals of the acute study. All data represent the mean ± SEM values for n = 3–8 animals per group (details of group sizes are presented in figure legends). The data were tested by one-way ANOVA for differences between age groups (19, 22, 31, 34 weeks) and no statistically significant differences were seen. Our main conclusions are not sensitive to the differences in age between the two exposure regimens. Differences between treatment groups were then examined by one-way ANOVA for the acute exposure with factor Dose and levels 0, 0.5, 2 Gy, and for chronic exposure with factor Dose and levels 0, 2 Gy, as well as two-way ANOVA with Dose (0, 2 Gy) and Time (acute regimen, chronic regimen) as factors. Tukey’s multiple comparison procedure was applied to elucidate patterns of significant effects (α = 0.05). Associations among the different endpoints were assessed by Pearson product moment correlation analysis.

## Results

There were no observable changes in general health status and for the parameters body weight, liver weight and spleen weight. For instance, the body weight values (g) were (mean ± SD, n = 15) 20.7±2.1 g (acute, control, 19 weeks old), 20.1±1.1 g (acute, 0.5 Gy), 20.6±1.4 g (acute, 2 Gy), 20.8±1.0 g (chronic, control, 22 weeks old) and 21.2±1.1 g (chronic, 2 Gy). Similarly, liver weight (% body weight) were 4.6±0.3, 4.7±0.3, 4.5±0.2, 4.6±0.3 and 4.5±0.2 respectively, while spleen weight (% body weight) were 0.44±0.05, 0.43±0.04, 0.41±0.06, 0.43±0.05 and 0.47±0.06 respectively.

### Oxidative Stress

**Figure 1:**
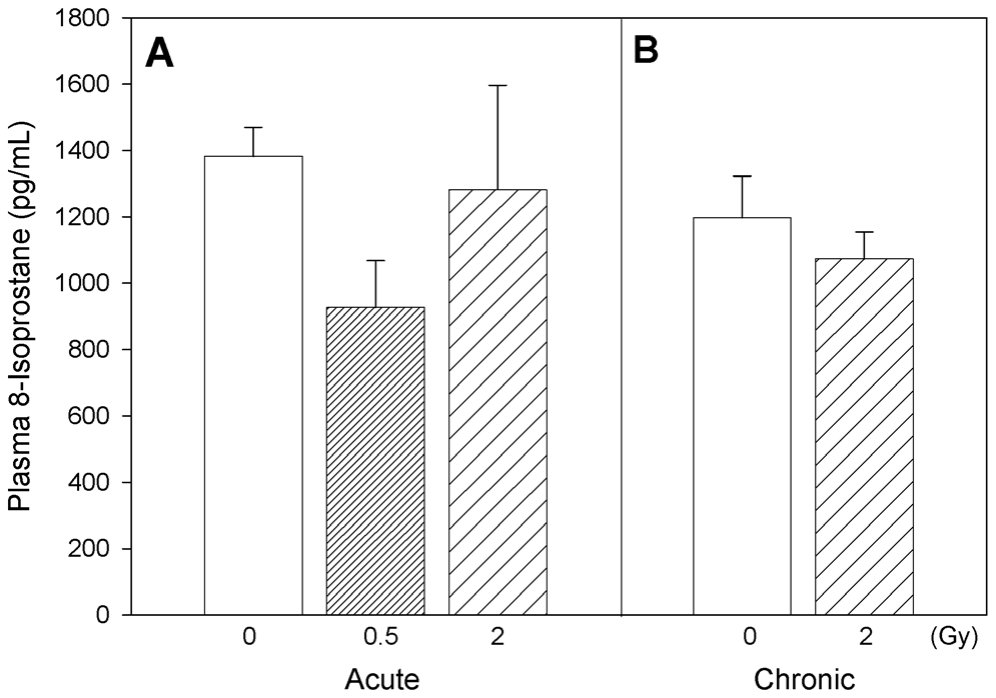
Marker of lipid oxidation. Plasma levels of 8–isoprostane in Apo-E−/− mice following acute (A) and chronic (B) radiation exposure. Mean ± SEM. Acute, 0 (n = 3), 0.5 Gy (n = 4), 2 Gy (n = 4). Chronic, 0 (n = 3), 2 Gy (n = 4).

**Figure 2:**
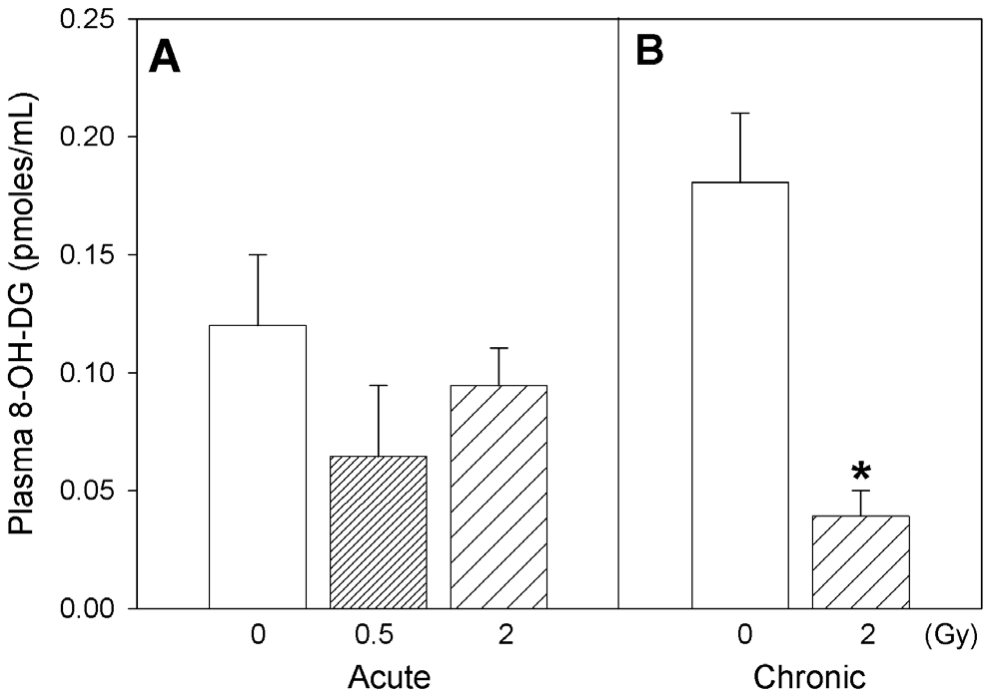
Marker of DNA oxidation. Plasma levels of 8-hydroxy-deoxyguanosine in Apo-E−/− mice following (A) acute (B) chronic radiation exposure. Mean ± SEM. Acute, 0 (n = 3), 0.5 Gy (n = 8), 2 Gy (n = 4). Chronic, 0 (n = 4), 2 Gy (n = 4). Two-way ANOVA with *Dose* (0, 2 Gy) and *Time* (acute, chronic) as factors. *Dose x Time* interaction, p = 0.047. * Tukey test, *Dose* within chronic, 0 vs 2 Gy, p<0.05.

Plasmatic oxidative stress-related changes were followed by measurement of oxidatively modified lipids, proteins, DNA and nitrated protein levels. ApoE−/− mice acutely exposed to 0.5 Gy radiation exhibited comparably lower 8-isoprostane levels (30% decrease, not statistically significant) in plasma (Figure 1A) after the post-radiation recovery period, while mice chronically exposed to 2 Gy radiation showed almost similar levels as of their corresponding controls (Figure 1B). No statistically-significant differences were detected in the levels of plasma 8-hydroxy deoxyguanosine, a marker of DNA damage, in mice acutely exposed to Co 60 (γ) (Figure 2A). However, the 8-OHDG levels were significantly decreased in plasma after repeated radiation exposure of the animals at 2 Gy recovery compared to corresponding control ApoE−/− mice (Figure 2B; two-way ANOVA, *Dose x Time* interaction, p = 0.047; Tukey’s, *Dose* within chronic exposure, 0 vs. 2 Gy, p<0.05).

**Figure 3:**
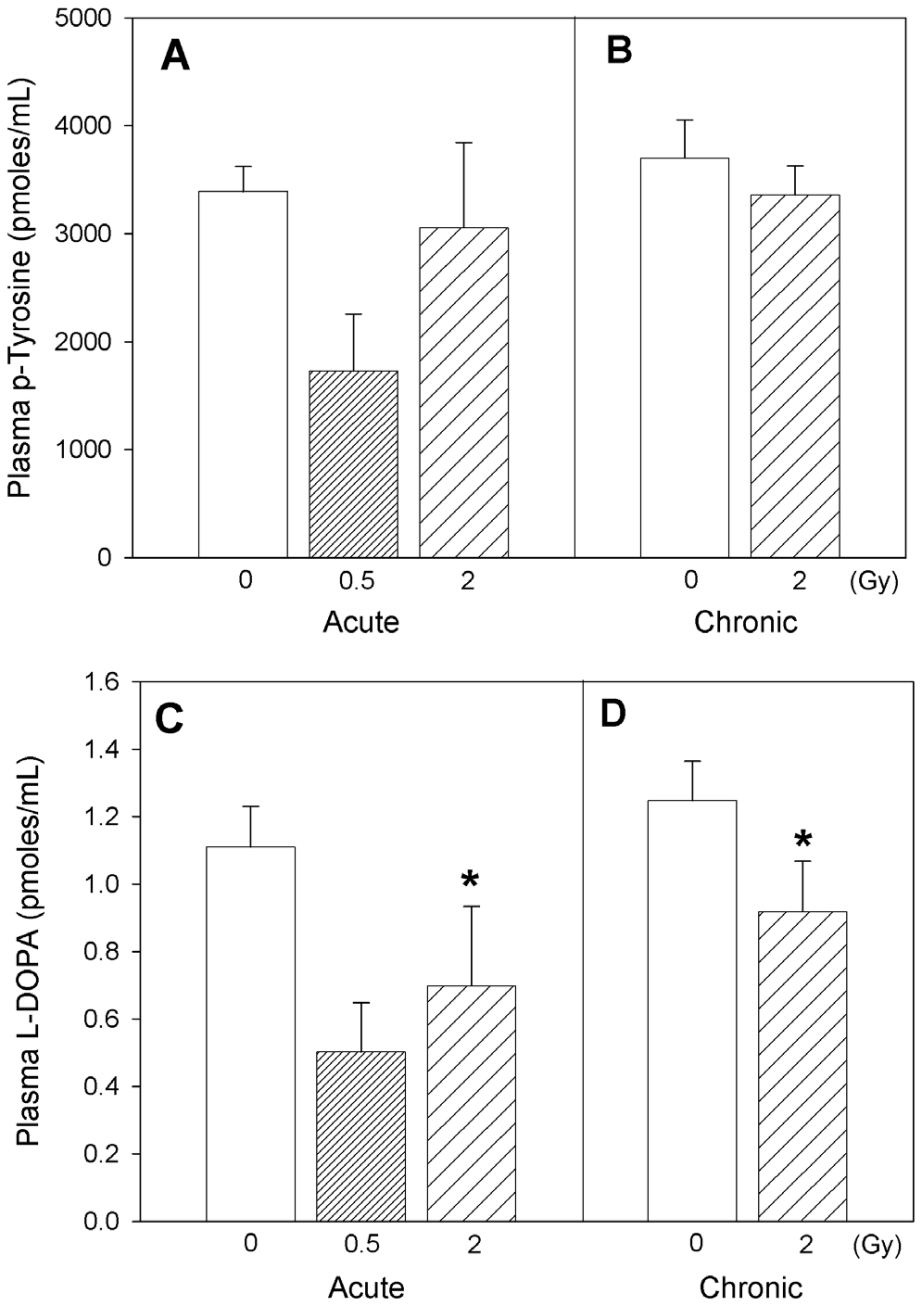
Markers of protein oxidation. p-Tyrosine (A, B) and L-Dopa (C, D) levels in plasma of Apo-E−/− mice following acute (A, C) and chronic (B, D) radiation exposure. Mean ± SEM. Acute, 0 (n = 3), 0.5 Gy (n = 8), 2 Gy (n = 4). Chronic, 0 (n = 4), 2 Gy (n = 4). L-DOPA: Two-way ANOVA with *Dose* (0, 2 Gy) and *Time* (acute, chronic) as factors, *Dose* main effect, p = 0.005. *Tukey test, 0 vs 2 Gy, p<0.05.

**Figure 4:**
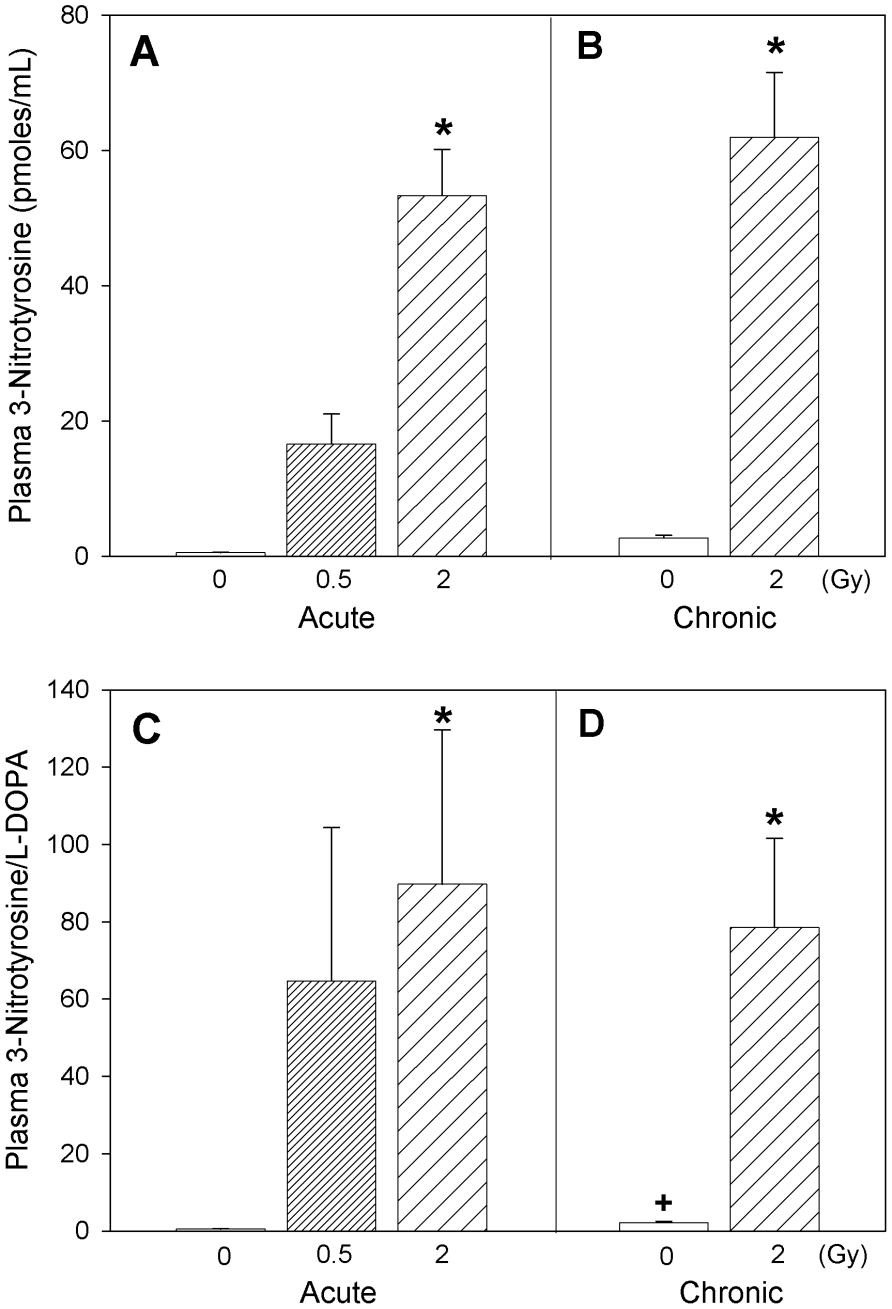
Marker of protein nitration. Plasma levels of 3-nitrotyrosine (A, B) and 3-nitrotyrosine/L-Dopa ratio (C, D) in Apo-E−/− mice following acute (A, C) or chronic (B, D) radiation exposure. Mean ± SEM. Acute, 0 (n = 3), 0.5 Gy (n = 8), 2 Gy (n = 4). Chronic, 0 (n = 4), 2 Gy (n = 4). 3-nitrotyrosine: Two-way ANOVA with *Dose* (0, 2 Gy) and *Time* (acute, chronic) as factors, *Dose* main effect, p = 0.004. *Tukey test, 0 vs 2 Gy, p<0.05. 3-nitrotyrosine/L-DOPA: Two-way ANOVA with *Dose* (0, 2 Gy) and *Time* (acute, chronic) as factors, *Dose* X *Time,* p = 0.025. *Tukey test, 0 vs 2 Gy, p<0.05. +Tukey test, acute vs chronic, p<0.05.

Changes in plasma levels of p-tyrosine, formed as a result of phenylalanine hydroxylation, did not show any statistically significant difference attributable to radiation exposure (Figures 3A–B), while L-DOPA, a marker of hydroxylation of p-tyrosine, decreased after radiation exposure (Figures 3C–D; two-way ANOVA, *Dose* main effect, p = 0.005; Tukey’s, 0 vs. 2 Gy, p<0.05). On the other hand, plasma profiles for 3-nitrotyrosine, a marker of protein nitration, markedly increased with acute (Figure 4A; one-way ANOVA on acute exposure data, p = 0.011; Tukey’s, 0 vs. 2 Gy, p<0.05) as well as chronic radiation exposure (Figure 4B; one-way ANOVA on chronic exposure data, p<0.001; Tukey’s, 0 vs. 2 Gy, p<0.05). Two-way ANOVA analysis of the 3-nitrotyrosine data with *Dose* (0, 2 Gy) and *Time* (acute, chronic) as factors exhibited a *Dose* main effect, p = 0.004 (Tukey’s, 0 vs. 2 Gy, p<0.05). The ratio of 3-nitrotyrosine to L-DOPA, an estimate of the competition between the nitrative and oxidative reaction pathways (Figures 4C–D), when tested by two-way ANOVA with *Dose* (0, 2 Gy) and *Time* (acute, chronic) as factors, revealed a radiation *Dose* main effect, p<0.001 (Tukey’s, 0 vs. 2 Gy, p<0.05), and a *Time* main effect (Tukey’s, acute vs. chronic, p<0.05). The small difference between the pooled 0 and 2 Gy animals from the acute regimen and the chronic regimen is attributable to the age difference between the two animal cohorts (*Time* main effect), but with no interaction with the *Dose* factor. The effects of radiation are not confounded by age.

### Vascular Markers

**Figure 5:**
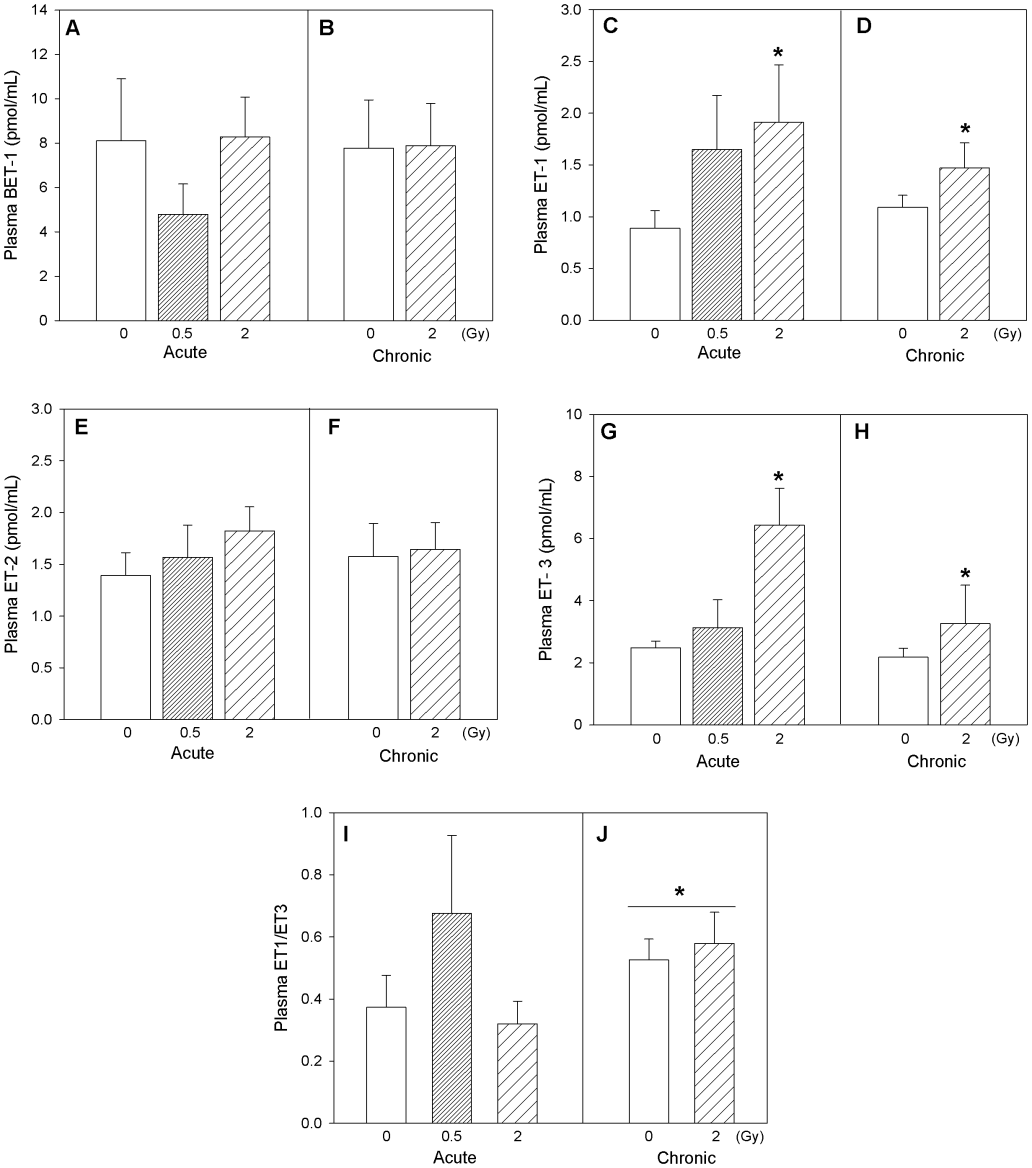
Vasoconstrictor peptides. Vasoconstrictor peptides BET-1 (A, B), ET-1 (C, D), ET-2 (E, F), ET-3 (G, H) and the ET-1/ET-3 ratio (I, J) in Apo-E−/− mice after acute (A, C, E, G, I) and chronic (B, D, F, H, J) radiation exposure. Mean ± SEM. Acute, 0 (n = 3), 0.5 Gy (n = 5), 2 Gy (n = 5). Chronic, 0 (n = 6), 2 Gy (n = 5). Two-way ANOVA with *Dose* (0, 2 Gy) and *Time* (acute, chronic) as factors. ET-1: *Dose* main effect, p = 0.016. *Tukey test, 0 vs 2 Gy, p<0.05. ET-3: *Dose* main effect, p = 0.020. *Tukey test, 0 vs 2 Gy, p<0.05. ET-1/ET-3: *Time* main effect, p = 0.032. *Tukey test, acute vs chronic, p<0.05.

**Figure 6:**
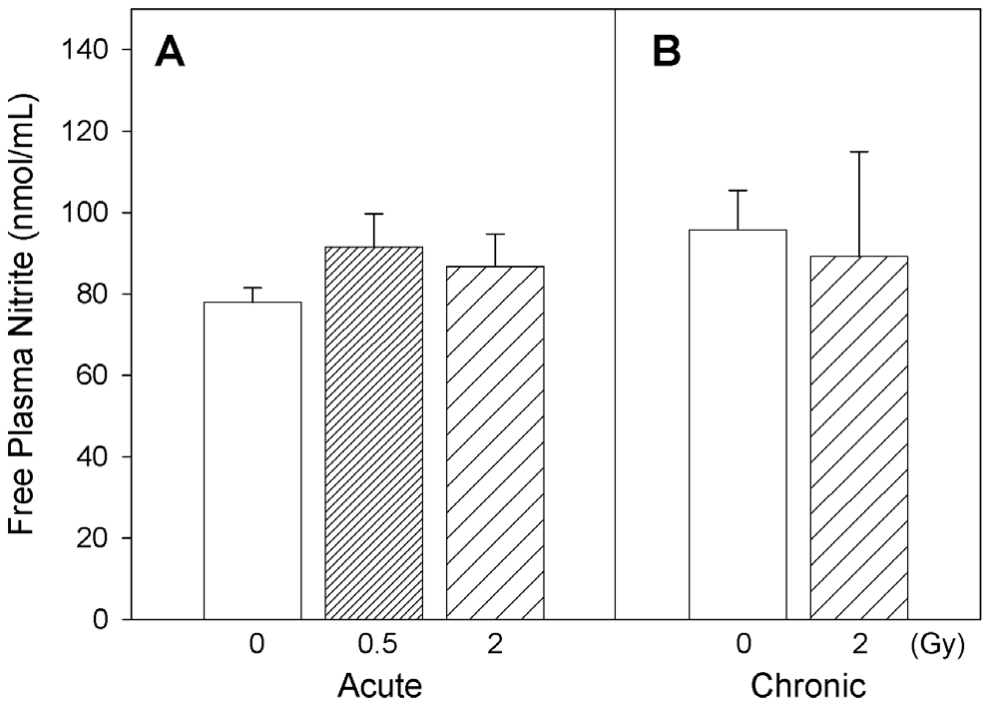
Marker of nitric oxide. Effect of acute (A) and chronic (B) radiation exposure on circulating nitrite levels in Apo-E−/− mice. Mean ± SEM. Acute, 0 (n = 4), 0.5 Gy (n = 7), 2 Gy (n = 3). Chronic, 0 (n = 4), 2 Gy (n = 3).

**Figure 7:**
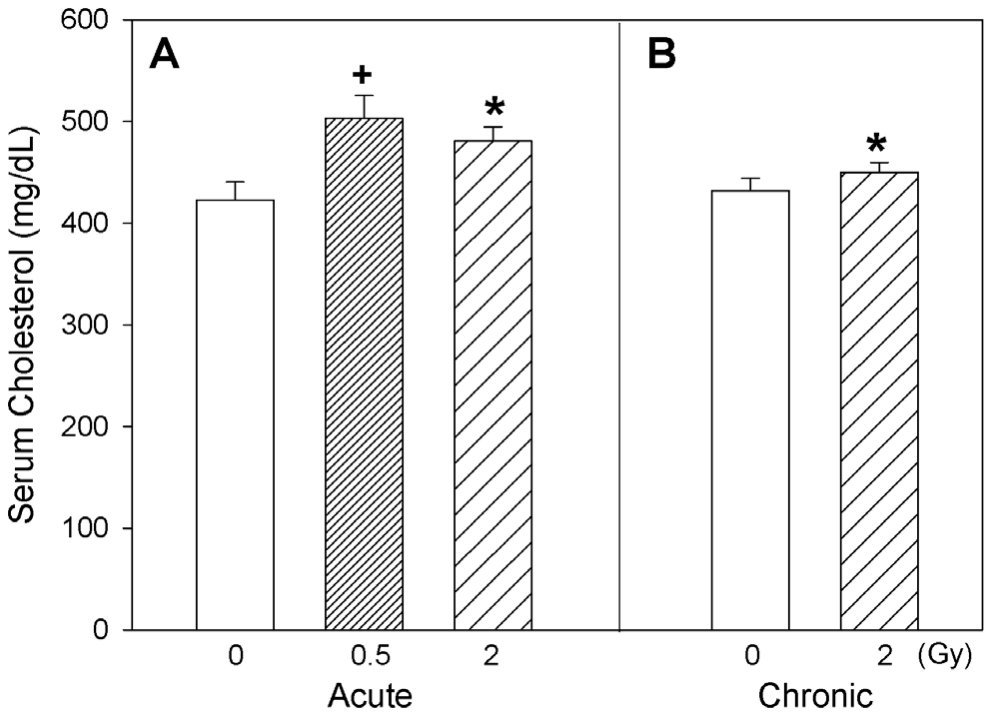
Blood cholesterol. Apo-E−/− mice serum cholesterol levels after acute (A) and chronic (B) exposure to radiation. Mean ± SEM. Acute, 0 (n = 15), 0.5 Gy (n = 15), 2 Gy (n = 15). Chronic, 0 (n = 15), 2 Gy (n = 15). One-way ANOVA on acute exposure (0, 0.5, 2 Gy), p = 0.011. +Tukey, 0 vs 0.5 Gy, p<0.05. Two-way ANOVA with *Dose* (0, 2 Gy) and *Time* (acute, chronic) as factors, *Dose* main effect, p = 0.008. *Tukey test, 0 vs 2 Gy, p<0.05.

Changes relevant to endothelial injury were assessed in this study by determination of circulating vasoconstrictor peptides and plasma nitrite levels. Circulating endothelin isoform profiles are shown in Figure 5A–H. In general, a similarity in the patterns of profiles is noticeable for ET-1, ET-2, ET-3 isoforms both under acute and chronic exposure scenarios. When mice were acutely exposed to radiation, ET-3 isoform exhibited significant increase in plasma at the highest dose (2 Gy) of exposure compared to the control and 0.5 Gy exposure groups (one-way ANOVA, p = 0.010). Overall analysis of radiation exposures on circulating ET isoforms (2-way ANOVA with *Dose* [0, 2 Gy] and *Time* [acute, chronic] as factors) exhibited significant increases in plasma ET-1 (p = 0.016) and ET-3 (p = 0.020) with radiation exposure, and increased plasma ET-1/ET-3 ratio (p = 0.032) with time (Figure 5I–J). Circulating nitrite levels suggested an increasing trend with acute exposure but no observable changes were seen with chronic radiation exposure (Figure 6A–B). In mice acutely exposed to 0.5 Gy radiation, serum cholesterol levels were significantly increased (one-way ANOVA, p = 0.011) compared to the corresponding controls (Figure 7A). Also, serum cholesterol levels were elevated after exposure to 2 Gy radiation, irrespective of whether the animals were acutely or chronically exposed (two-way ANOVA, *Dose* main effect, p = 0.008; Figure 7A–B).

**Figure 8:**
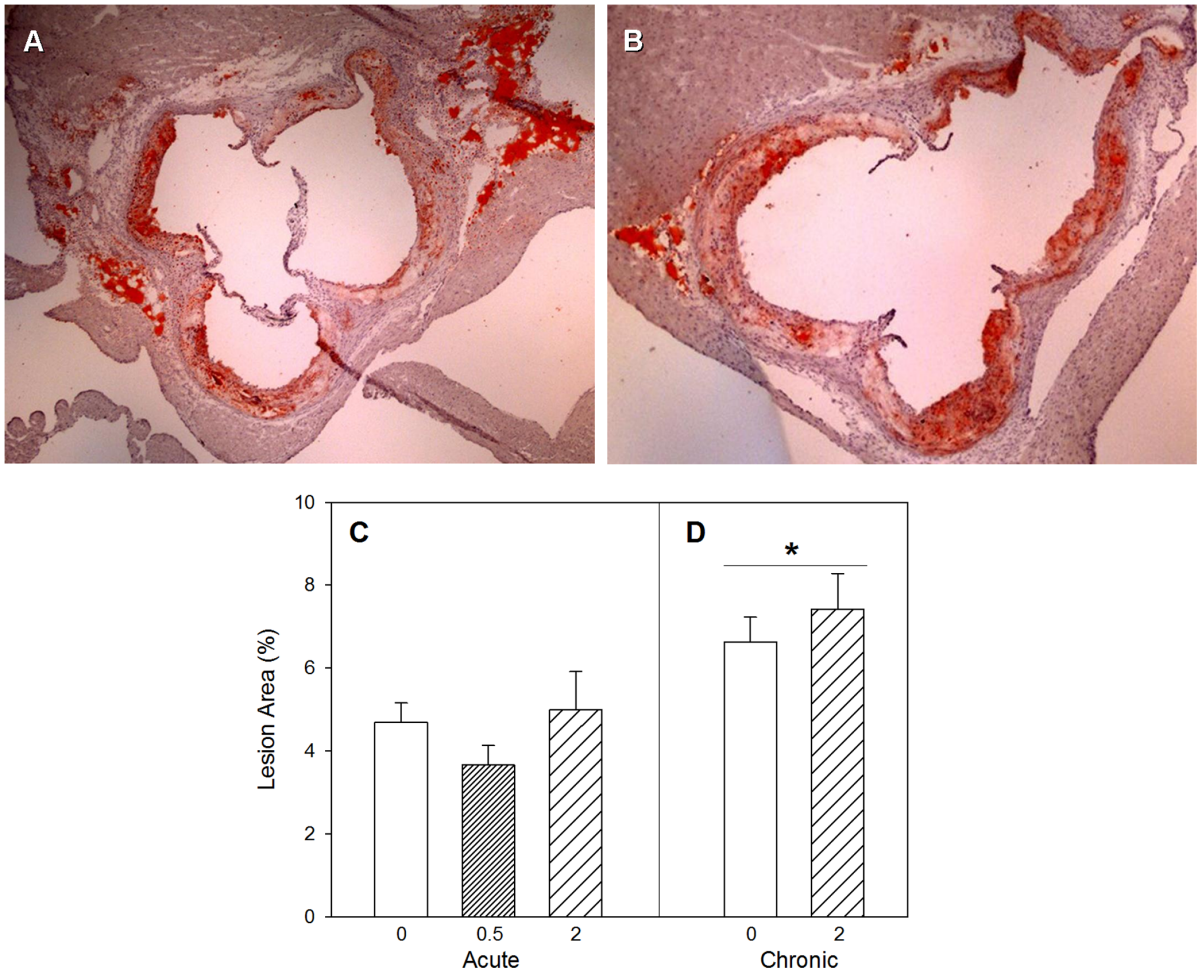
Atherosclerosis plaques. Sections of the orifices of the coronary arteries marking the start of the ascending arch in a 5-month old control ApoE−/− mouse (A) and an ApoE−/− mouse following chronic radiation exposure (B). Percent lesion area profiles for acute (C) and chronic (D) radiation exposure. Mean ± SEM. Acute, 0 (n = 15), 0.5 Gy (n = 15), 2 Gy (n = 15). Chronic, 0 (n = 15), 2 Gy (n = 15). Two-way ANOVA with *Dose* (0, 2 Gy) and *Time* (acute, chronic) as factors, *Time* main effect, p = 0.005. *Tukey test, acute vs chronic, p<0.05.

Sections of the orifices of the coronary arteries marking the start of the ascending arch confirmed lesions in both chronic control and 2 Gy exposed groups (Figure 8A–B). Determination of percent lesion area revealed a progression of atherosclerosis (Figure 8C–D) attributable to an age difference of 2–3 weeks between the animals of the acute cohort and those of the chronic cohort (two-way ANOVA, *Time* main effect, p = 0.005). Radiation treatment effects such as the 25% decrease of lesion in 0.5 Gy animals of the acute exposure and the 15–20% increase of lesion in the 2 Gy animals of the chronic study were not statistically significant (p>0.05).

## Discussion

There is increasing evidence that exposure to ionizing radiation may trigger initiation or modulation of cardiovascular diseases [9], [19]. In addition, analysis of Japanese A-bomb survivor data suggests that non-cancer (including heart disease, stroke, digestive and respiratory diseases) mortality can contribute almost equally to cancer mortality and thus can be associated with radiogenic excess risk [35]. However, there is a controversy whether such effects occur due to low or high doses of exposure [10], [28], [36], [37]. It is essential to identify biological mechanisms that can explain low-level radiation effects, including reported potential protection effects, to determine whether this is true for all cardiovascular processes and disease stages.

We have reported previously protective effects of acute radiation exposures at low doses delivered at low dose rates, while at high dose rates both protective and detrimental effects have been observed in ApoE-deficient mice [28]. In this study, we exposed ApoE-deficient mice to radiation acutely and chronically to investigate toxicity mechanisms relevant to cardiovascular effects. For chronic exposures at 2 Gy, mice were exposed at 0.1 cGy/min, a dose rate selected on the basis of protective effects against chromosomal breaks in human cells [38], neoplastic transformation in mouse cells [39], and radiation-induced myeloid leukemia in mice [40].

Apolipoprotein E acts as the main factor in the mechanism for removal of cholesterol enriched chylomicrons and remnants of very low density lipoproteins [41]. Transgenic mice deficient in apolipoprotein E can serve as a genetic susceptibility model for cardiovascular disease because when fed a normal low fat diet they are susceptible to atherosclerosis, and on high fat diet the progression of the disease is known to be exacerbated.

Our results revealed that metabolites related to reactive oxygen species (ROS) formation, irrespective of whether they were formed due to oxidative transformation of lipid (8-isoprostane), protein (p-tyrosine, L-DOPA) or DNA (8-hydroxydeoxyguanosine), displayed similar relative responses to radiation exposure (Figures 1–3). It is also interesting to note that assessment of association between L-DOPA, p-tyrosine and 8-OH-DG in plasma exhibited significant (p<0.05) positive correlations within the experimental animals (Pearson Product Moment correlation analysis), confirming the commonality in their ROS-dependent formation. Interestingly, our results indicated that radiation exposures led to decreased circulating ROS biomarker levels several months after radiation treatment, despite the well established role of ROS generation during radiation exposure. The reduction of ROS after recovery can be interpreted as a protective effect. Nevertheless, the levels of plasma 3-nitrotyrosine in these mice revealed that radiation exposure enhanced the rate of reactive nitrogen species (RNS) formation significantly, several months after a single exposure (p = 0.001) or repeated low-dose rate exposures (p = 0.004) (Figure 4A–B). The relationship between 3-nitrotyrosine levels and cumulative dose was linear (p<0.001).

It is also noteworthy that plasmatic ROS-generated products p-tyrosine, L-DOPA and 8-OH-DG levels exhibited a statistically significant negative association (Pearson Product Moment correlation, p<0.05) with 3-nitrotyrosine levels in individual animals, supporting the concept of competition between oxidative and nitrative reaction pathways *in vivo*. The ratio of 3-nitrotyrosine to L-DOPA, which reflects an estimate of the competition between nitrative and oxidative reaction pathways, increased under both acute and chronic radiation exposure conditions (Figure 4C–D), and also exhibited a linear dose-response relationship with radiation exposure (p = 0.022). It is well known that ionizing radiation affects biological systems through formation of reactive oxygen species [42]. Induction of superoxide (O_2_^.−^) formation and increased release of NO^.^ species in atherosclerosis [43], could have reacted to form peroxynitrite leading to the formation of 3-nitrotyrosine [44]. Therefore, persistent nitrative stress may be a long-term and possibly progressive consequence of radiation exposure.

Formation of peroxynitrite has been reported in atherosclerotic lesions and is implicated in vascular injury [43]. Similarly, 3-nitrotyrosine and nitrated proteins are implicated in various diseases including tissue injury especially in severity of cardiovascular diseases, myocardial inflammation and endothelial dysfunction [45], [46]. Our findings of radiation exposure-induced increase of the circulating 3-nitrotyrosine levels can thus reflect a persistent exacerbation of nitrative stress and thus inflammation in atherosclerotic plaques. These results suggest that additional analyses of the nitroproteome may reveal additional biomarkers of radiation exposure-induced cardiovascular changes. Meanwhile, free circulating plasma nitrite levels (Figure 6A–B) did not exhibit much change attributable to radiation exposure, suggesting that perturbation of nitric oxide production in the general vasculature, if any, was reversed after recovery of the animals from acute or chronic radiation.

Circulating levels of the vasoactive peptides endothelins, ET-1 and ET-3 increased with acute and chronic radiation exposures, also suggesting changes in the cardiovascular system. Similar to 3-nitrotyrosine, the ET-1 (p = 0.134) and ET-3 (p = 0.016) isoforms exhibited linear dose-related increases in plasma with increasing cumulative dose of radiation (Figure 5A–H). The ET-1/ET-3 ratio, which has been reported to be a better prognostic indicator for pulmonary hypertension compared to ET-1 alone [47], was elevated in animals from the chronic exposure cohort (control and radiation-exposed animals) by comparison to the animals of the acute exposure cohort, revealing a shift of ET-1/ET-3 with progression of atherosclerosis. Endothelin-1 is a prognostic indicator for cardiovascular diseases and is elevated in atherosclerosis due to the increased expression of endothelin converting enzyme (ECE) in plaques, which accelerates conversion of the precursor big ET-1 to ET-1 [48]. Endothelin-1 is also mitogenic and advances atherosclerotic plaque formation and growth [49]. Endothelin-3 is implicated in pulmonary artery hypertension and in affecting systemic blood pressure transiently [50], [51]. It is also known that ET-3 is a ligand of the ET_B_ receptor and can cause vasodilation through induction of NO production [52]. The parallel increase in circulating ET-1 and ET-3 levels imply that there may be compensatory mechanisms with radiation exposure counteracting the vasopressor effects of ET-1.

Because BET-1 was not modified by radiation exposure, the elevation in circulating ET-1 does not appear to relate to a general transcriptional activation of vascular preproendothelin-1 gene with increased *de novo* synthesis, but more logically to an increase in the rate of conversion of BET-1 to ET-1 by ECE, which is consistent with plaque activation. It is well documented that elevated ECE in atherosclerotic plaques promotes the conversion of circulating BET-1 [53]. Long term up-regulation of the endothelin system can have detrimental effects due to the vasopressor, pro-fibrotic, and pro-hypertrophic properties of ET-1 [54]. Our observations support the notion that exposure to ionizing radiation can modulate the endothelinergic system, which may be a basic mechanism underlying radiation-induced cardiovascular effects. These findings are in line with delayed enhancement of cardiac fibrosis after radiation exposure of ApoE-deficient mice [29].

Although serum cholesterol levels were already high in the control animals due to their genetic deficiency, our results on total serum cholesterol levels indicated an increase (p<0.05) with radiation exposure (Figure 7A–B). This observation is in line with a previous report of an association between radiation exposure and increased total serum cholesterol in A-bomb survivors [55]. Also, the observation of increased cholesterol level in the mice acutely exposed to 0.5 Gy is consistent with our previous observations [28]. Increase in total serum cholesterol levels due to radiation exposure in the ApoE-deficient mice can perhaps be attributed to impairment in the ability to metabolize and clear serum lipids. The statistically significant, age-dependent and radiation-independent increase in percent lesion area is attributed to progression of atherosclerosis, which is particularly steep between 4 months and 6 months of age in the ApoE-deficient mice. Nevertheless, the non-significant decrease in percent lesion area at acute 0.5 Gy exposure is in line with a protective effect described previously [28].

Among the endpoints assessed in this work, both plasma levels of 3-nitrotyrosine and endothelin isoforms exhibited a linear association with cumulative radiation dose. Increase in plasmatic 3-nitrotyrosine has been associated before with vascular injury [23], while rise in circulating levels of ET-1 has been linked with endothelial dysfunction [49]. In a previous report, vascular injury and endothelial dysfunction due to modulation of renin-angiotensin and endothelinergic systems were implicated in radiation exposure-induced heart disease [54]. These observations are in line with our findings. Although we did not measure statistically significant increase in percent lesion area after repeated exposure to radiation at the 0.1 cGy/min dose-rate, the increase in plasmatic ET-1 and 3-nitrotyrosine levels suggest activation of the lesion biology by radiation exposure and could be a factor in delayed cardiac diseases [29].

### Conclusion

Although ROS formation is one of the key mechanisms known to be triggered by radiation exposure, in atherosclerosis disease process, recovery from exposure to radiation appears to preferentially promote RNS pathway and formation of 3-nitrotyrosine. Similarly, enhanced plasmatic ET-1 and ET-3 imply that radiation exposure can exacerbate cardiovascular changes relevant to endothelial dysfunction and thus disease progression. At acute low dose (0.5 Gy) radiation delivered at the high dose rate, the decrease in ROS products and percent lesion area can imply protective effects, yet the circulating 3-nitrotyrosine, ET-1 and cholesterol levels suggest detrimental effects. Whereas, at chronic (2 Gy) radiation exposure, more detrimental effects are seen in terms of circulating 3-nitrotyrosine, ET-1 and cholesterol levels, as well as percent lesion area. Our observations in an animal model, under experimental conditions, imply a linear dose-response relationship with no threshold for radiation-induced cardiovascular effects, i.e. nitrative stress and elevation of steady-state ET-1 circulating levels. Proteomic investigation of the molecular targets of nitrative stress and a network analysis anchored on the endothelinergic system may help to identify and verify valuable biomarkers for elucidation of radiation-induced cardiovascular diseases in patients and human populations. Better understanding of radiation-exposure toxicity pathways would enhance the assessment of human health effects of low level radiation and the development of targeted intervention strategies in prevention of health outcomes, especially in cases of radiotherapy-induced heart disease.
